# Serum Cholesterol Reduction Efficacy of Biscuits with Added Plant Stanol Ester

**DOI:** 10.1155/2015/353164

**Published:** 2015-03-10

**Authors:** Wantanee Kriengsinyos, Ajima Wangtong, Surat Komindr

**Affiliations:** ^1^Institute of Nutrition, Mahidol University, Salaya, Nakhon-Pathom 73170, Thailand; ^2^Faculty of Medicine, Ramathibodi Hospital, Mahidol University, Bangkok 10400, Thailand

## Abstract

This study's aim was to test the low-density lipoprotein cholesterol- (LDL-c-) lowering efficacy of biscuits containing 2 g of plant stanols, which corresponded to 3.4 g of plant stanol esters. The biscuit is a new food format that can be consumed as a snack. In a double-blind, placebo-controlled parallel design study, 119 mildly to moderately hypercholesterolemic volunteers were randomized to plant stanol or control groups. Subjects were comparable in age, gender, lipid profiles, and body mass index. They consumed a control biscuit once a day for a two-week period, followed by a four-week intervention period that either had a plant stanol ester biscuit or a control. During the habitual diet, one biscuit per day was consumed at any time that subjects wished. Serum lipid profiles were measured at the first day of run-in, at baseline, and at the study's end. Compared to the control, the total cholesterol (TC), LDL-c, and the LDL-to-high-density lipoprotein (LDL/HDL) ratio had serum reductions of 4.9%, 6.1%, and 4.3%, respectively, and were observed after 4 weeks of biscuit consumption with added plant stanols (*P* < 0.05). A significantly higher reduction in LDL-c (8.9%) and LDL/HDL ratio (11.4%) was measured in those taking a plant stanol biscuit with a meal compared to those who consumed a plant stanol biscuit without other food. In conclusion, incorporating plant stanols into a biscuit is an attractive, convenient, and acceptable way to modestly lower elevated cholesterol concentrations. For optimal efficacy, biscuits should be consumed with a meal as part of a healthy diet.

## 1. Introduction

Association of high blood cholesterol with the development of atherosclerosis and coronary heart disease is well established [1, 2]. A high prevalence of hypercholesterolemia exists throughout the world including Thailand. Based on the Fourth Thai National Health Examination Survey in 2009, the prevalence of high low-density lipoprotein cholesterol (LDL-c) levels was 29.6% [3]. This rate was double to one-third that reported in the previous third survey conducted in 2004 (14% of men and 17% of women) [4]. Therapeutic lifestyle changes (reduced dietary intake of saturated fats and cholesterol and weight control and increased physical activity) form the core of all cholesterol-lowering initiatives. The National Cholesterol Education Program (NCEP) [5] and the American Heart Association recommend supplemental therapy with plant sterols and stanols (phytosterols or phytostanols) as a dietary adjunct for individuals who do not achieve their LDL-c treatment targets through diet alone [6].

The cholesterol-lowering properties of plant sterols have been known since the 1950s [7]. Plant sterols decrease the absorption of cholesterol from the small intestine [8]. The saturated form of plant sterols, termed plant stanols, decreases serum levels of both cholesterol and plant sterols, and they are not absorbed to any significant degree [9–13]. Fat-soluble plant stanol esters have been developed as ingredients in food products to achieve a significant reduction in serum total and LDL-c levels. In 2009, the European Food Safety Authority Panel on Dietetic Products, Nutrition and Allergies concluded that plant stanol esters, at a daily intake of 1.5–2.4 g/d, can lower LDL-cholesterol by 7.0 to 10.5%. The minimum duration required to achieve the maximum effect of LDL-cholesterol-lowering is two to three weeks [14].

Over 60 clinical studies have confirmed the cholesterol-lowering efficacy of plant stanol esters [15–17]. Traditional plant stanol ester food products that are marketed include margarine, spreads, salad dressings, and shortenings (high fat products) [12, 18–23], as well as some desserts such as yogurt- or soy-based mini drinks (low fat products) [24–27]. In most clinical studies, cholesterol-lowering efficacy was demonstrated when plant stanol esters were incorporated into mayonnaise, low fat-spreads, or yogurt [17, 28]. In a recent meta-analysis, LDL cholesterol values were reduced by 9 percent through a 2 gram daily dose of plant stanols. Moreover, increasing the daily intake of plant stanols was found to dose-dependently reduce LDL cholesterol [17]. Plant stanol esters reduce serum cholesterol by replacing dietary and biliary cholesterol in mixed micelles during digestion. Subsequently, less cholesterol is available for enterocytic absorption [24, 29].

Currently in Thailand, 3 out of 4 people aged 6 years and over regularly consume snacks; a rate that increased from 74.1% in 2009 to 77.6% in 2013 [30]. Eating snacks is classified as an unhealthy habit because most snacks, especially popular crispy snacks, are high in fat and energy. Consequently, a healthy snack is needed to promote better health. This study incorporated a newly added plant stanol into a popular and convenient food format, a biscuit, that can be consumed as a healthy snack. This study's purpose was to investigate the total and LDL cholesterol-lowering efficacy of biscuits with added plant stanols. It was expected that a biscuit's solid food matrix, which contains more fat and protein than a liquid matrix and with fast transit time, would facilitate necessary gastrointestinal digestive and absorptive processes and promote efficient cholesterol lowering properties.

## 2. Subjects and Methods

### 2.1. Subjects

In total, 213 volunteers were recruited via intranet and posters at the Salaya campus of Mahidol University, Thailand. All volunteers received telephone interviews. Sixty-eight persons were excluded because they did not meet the inclusion criteria. The remaining 145 subjects were invited for blood screening. The primary selection criteria for subjects were aged between 25 and 60 years; a fasting serum TC concentration of 5.2 to 6.5 mmol/L; fasting serum triglyceride levels below 3.0 mmol/L; absence of renal, diabetes, hepatic, thyroid, or alcohol problems; and pretest willingness to consume biscuits throughout the study period. Exclusion criteria included the use of cholesterol-lowering medication, plant stanol or plant sterol products or any supplements, pregnancy or lactation, alcohol or drug abuse, insulin-treated diabetes, or unstable coronary artery disease. On the basis of these criteria, 120 volunteers were accepted as study subjects. A sample size was determined to be 54 persons for each group, based on a previous study [21] showing a mean reduction of 10 percent of LDL cholesterol using plant sterols. An additional 10 percent in the number of subjects was added to allow for potential drop-outs; approximately 60 volunteers per group were needed. The study was conducted following the guidelines of Good Clinical Practice (ICH-GCP). Written informed consent was obtained from all study subjects.

### 2.2. Study Design

A randomized, double-blind, placebo-controlled study was conducted including a two-week run-in period, which entailed consumption of a control biscuit, followed by a four-week study period. The Clinical Research Unit at the Institute of Nutrition, Mahidol University, performed a parallel intervention study comparing biscuits with plant stanol ester (plant stanol group) with placebo biscuits (control group). The Ethics Committee of the Mahidol University Institutional Review Board (Registration number 2013/004.1001) approved the study, which was registered as part of Clinical Trials (NCT02331043).

After stratification by gender and total cholesterol levels, subjects were randomized to plant stanol and control groups. They were advised to consume one biscuit per day, while maintaining their habitual diet, physical activity, and lifestyle. Subjects could consume the biscuit at any time they wished. The time of biscuit consumption was recorded and specified as before, between, or after meals. If the biscuit was consumed between meals, any food that was consumed with the biscuit was also recorded. A meal was defined as any food or drink that provided together with the intervention biscuit more than 920 kJ. The placebo and experimental biscuits were similar in color, taste, fat content, and caloric value to preserve the double-blind study design. A three-digit code number was used for each product. The code numbers were opened after all analyses were performed. Raisio Nutrition Ltd. (Raisio, Finland) provided the test biscuits. The biscuits came in two flavors: chocolate and fruity. The nutritive value of the biscuits and the composition of the plant stanol mixture are shown in Table 1. The biscuits were distributed every two weeks, at which time compliance and side effects were recorded through a questionnaire.

### 2.3. Anthropometric Measurement and Diet Record

Each subject's height was measured using a stadiometer to the nearest 0.1 cm. Body weight and body composition were measured using bioelectrical impedance analysis (BIA) (Tanita BC-545, Japan) while the subject stood without shoes/socks and dressed in indoor clothing. Body weight and body composition were measured at the beginning, at the middle, and at the end of the intervention study. Volunteers were asked to record the type and amount of all foods consumed on Thursday, Friday, and Saturday or Sunday prior to the run-in period, prior to the intervention period, as well as in the middle and at the end of the intervention period. Dietitians trained the subjects to use scales, measuring spoons, and cups to estimate portion sizes. At each submission, dietary records were reviewed to resolve any uncertainty in the entries and to assess the completeness of the records. The completed diet records were evaluated for energy, carbohydrate, protein, and fat intakes, using the INMUCAL software program (version NB.1.1, Institute of Nutrition, Mahidol University, Thailand).

### 2.4. Biochemical Indicators

Twelve-hour-fasting blood samples were taken before the study began (run-in), baseline (at the end of week 2), and at the end of the intervention study (week 6). All samples (weeks 0, 2, and 6) were measured for serum total cholesterol (TC), low-density lipoprotein cholesterol, high density lipoprotein cholesterol (HDL-c), and triglyceride (TG). Concentrations of lipid parameters were analyzed by enzymatic colorimetric methods using an Olympus/AU 400 auto analyzer. Interassay variability was 3.24% for TC, 1.11% for LDL-c, 0.75% for HDL-c, and 1.09% for triglycerides. Serum gamma-glutamyltransferase (GGT) and L-alanine aminotransferase (ALT) were measured by kinetic enzyme assay at baseline and at the end of study. The coefficient of variation for GTT and ALT was 1.29% and 1.63%, respectively.

### 2.5. Statistical Analyses

Normality and homogeneity of variance assumptions were evaluated before conducting detailed statistical analyses. A Student's *t*-test was used for comparing dietary intake between groups. The analysis of variance for repeated measurements (General linear model, GLM) was used to analyze the interaction of time and group, as well as changes over time in between-group comparisons followed by post hoc comparisons with Bonferroni corrections. All statistical analyses were performed with SPSS for Windows 13.0 statistics program (SPSS, Chicago, IL, USA). The results were presented as mean ± SD for continuous variables and proportion for categorical variables. A *P* value of less than 0.05 was considered significant.

## 3. Results

### 3.1. Subject Characteristics, Compliance, and Drop-Out Rate

One hundred nineteen out of 120 volunteers (59 in the pant stanol group and 60 in the control group) completed the study. During the study period, one volunteer in the plant stanol group was dropped due to consuming biscuits less than 85 percent of the time. The baseline characteristics of participants are shown in Table 2. There were no significant differences in age, gender ratio, BMI, and serum lipoprotein levels between the stanol and placebo groups, as well as between the sub-group of the stanol group that entailed taking a biscuit with or without meal. Compliance with the test products was excellent, since 99.2 and 99.4 percent of subjects in the plant stanol and control groups, respectively, consumed biscuits every day during the study period and experienced no side effects.

### 3.2. Dietary Intake and Body Weight

For both groups during the intervention, there were no changes in energy, carbohydrate, protein, and fat intakes. Consequently, the proportion of each nutrient to overall energy intake was not affected (Table 3). Physical activity and other lifestyle habits remained stable. Throughout the study, no significant changes in weight or waist circumference were observed.

### 3.3. Serum Lipids and Lipoproteins

**Figure 1:**
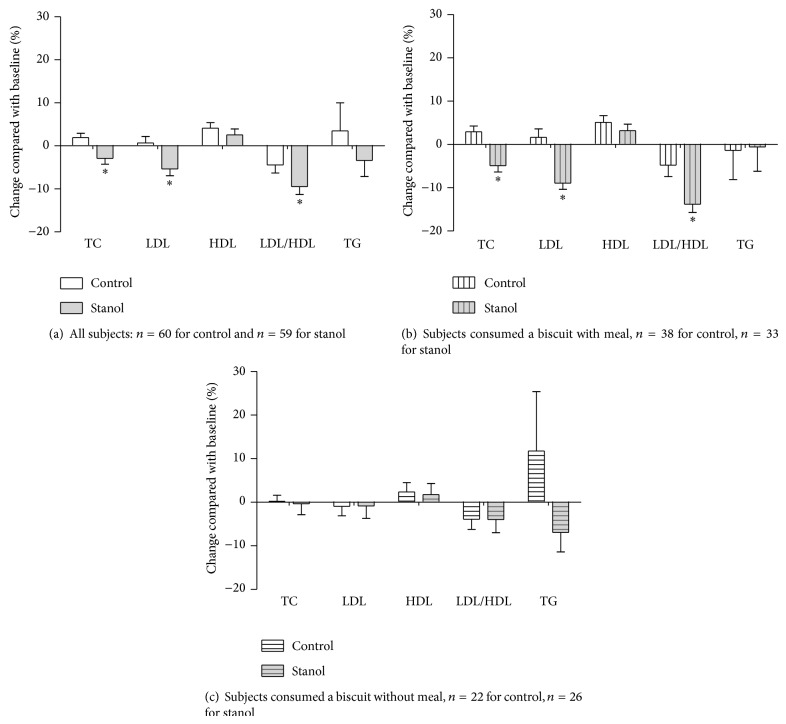
The percentage change in total, LDL, HDL, and LDL/HDL ratio and triglyceride concentrations from baseline comparing between control and stanol groups (mean ± SEM), (a) total subjects; (b) subgroup: subjects consumed a biscuit with meal; (c) subgroup: subjects consumed a biscuit alone (without meal). ^*^ *P* < 0.01 significantly different from control group.

In the plant stanol group, a significant reduction in the serum TC, LDL-c, and LDL/HDL ratio was observed at the end of study when compared to baseline: 2.9%, 5.4%, and 7.1%, respectively (Figure 1). The respective reductions were 4.9%, 6.1% and 4.3% compared to controls (*P* < 0.05). A trend in the reduction of serum TG in the plant stanol group was observed, but it did not reach significance.

Overall, 55.8% and 63.3% of subjects in the plant stanol and control groups, respectively, consumed biscuits daily with meals, mostly at lunch time. For the remaining participants, 44.2% and 36.7% in the plant stanol and control groups, respectively, consumed the biscuit alone without additional food. Serum LDL-c and LDL/HDL ratios for subjects who consumed biscuits with a meal or an energy-containing beverage decreased significantly over time in the plant stanol group (8.9% to 11.4%, resp., as shown in Figure 1(b)). No decrease in LDL-c and a small decrease (2.9%) in LDL/HDL ratios at the end of the study, compared to baseline, were observed in the control group. There was no change in LDL-c in the group that consumed the biscuit without a meal (Figure 1). No change in HDL-c and TG concentration was observed in subjects who consumed the biscuits with a meal or without a meal for within-group and between-group comparisons. The percentage change in serum lipids from baseline of the plant stanol group compared to subjects who consumed the biscuit with a meal or energy-containing beverage and without meal is shown in Table 4. The significantly higher efficacy in lowering TC (4.9%), LDL-c (8.9%), and LDL/HDL ratios (11.4%) was observed in subjects who consumed biscuits with plant stanols with a meal or energy-containing beverage, compared to a decrease of 0.9 percent in LDL-c and 1.8 percent in LDL/HDL ratios among subjects who consumed biscuits with plant stanols alone.

### 3.4. Liver Enzymes

Serum ALT and GGT concentrations increased significantly from baseline in both groups, but the changes did not differ between the groups (Table 5). There were 14 and 9 subjects in the plant stanol and control groups, respectively, who had elevated liver enzymes at baseline. When these subjects were excluded, significant increases in serum ALT and GGT remained from baseline in both groups; however, these increased levels remained in the reference range for healthy individuals.

## 4. Discussion

This study showed that serum TC and LDL-c concentrations after the four-week intervention were reduced significantly only in subjects who consumed plant stanols. A sub-analysis revealed that the cholesterol lowering efficacy of the biscuit with added plant stanol ester was dependent on whether the biscuit was consumed with or without a meal, with the best cholesterol-lowering efficacy demonstrated with a meal.

Daily consumption of a biscuit containing two grams of plant stanols for a period of four weeks decreased serum TC and LDL-c concentrations by 4.9% and 6.1%, respectively, compared to subjects in the control group who were mildly hypercholesterolemic and consumed a habitual Thai diet. The overall reduction in TC and LDL-c concentrations was lower than demonstrated in our previous study, which showed a reduction of 8.0% and 13.6% in the level of TC and LDL-c, after consuming plant stanol ester fortified soymilk (2 g plant stanols/d) [27]. In the present study, the same plant stanol ester dose (2 g plant stanols) was added into a different food matrix, the biscuits, instead of soymilk. In addition, stanol-added product consumption was most likely different between the studies. Subjects consuming soymilk with plant stanol ester were instructed to consume the soymilk with a meal (either breakfast or lunch). However, in the present study, biscuits with plant stanols were consumed at any time depending on each volunteer's preference. Plant stanols were reported to have the most cholesterol-lowering efficacy when taken as part of a meal, which resulted in an 8.9% decrease in LDL-c [23, 28, 31–33]. This decrease was associated with plant stanol ester, since there was no change in energy, carbohydrate, protein, and fat intakes during the intervention within both groups. The total energy intake of all subjects was around 5.65 MJ/day (1350 kcal/day) throughout the study, which may be somewhat underreported. However, this value was close to the average energy intake of 6.02 MJ/day (1440 kcal/d) (i.e., 6.87 MJ [1642 kcal] in males and 5.18 MJ [1238 kcal] in females among Thai adults aged 31–50 yrs.) as reported in the Fourth National Health Examination Survey, Food Consumption Survey of Thai Population (2008-2009) [34].

Mechanisms for cholesterol reduction are interference in cellular cholesterol metabolism within the enterocyte [35], inhibiting chylomicron formation by making cholesterol less available in intestinal cells [36], competing with cholesterol transporters, and for space in mixed micelles [37, 38]. Plant sterol addition to a meal displaces cholesterol from the intestinal aqueous phase and lowers chylomicron cholesterol occurrence in humans [39]. A plant sterol-containing yogurt drink, which was consumed before a meal, exhibited fast gastric emptying, with no triggering of gallbladder contraction, and in consequence did not inhibit cholesterol absorption [40]. In the present study, sub-analysis of the data found a higher efficacy in lowering LDL-c (decrease 8.9%) among hypercholesterolemic subjects who consumed stanol-fortified biscuits with a meal compared to only a 0.9% reduction in LDL-c when consuming stanol-fortified biscuits alone. These results were in line with a study by Doornbos et al. which investigated the impact of intake occasion (with or without a meal) and a food product's fat level impact on the cholesterol-lowering efficacy of a plant sterol-enriched (3 g/d) single-dose yogurt drink. They found that a substantially larger decrease in LDL-c concentrations was achieved when the single dose yogurt was consumed with a meal independent of its fat content [41].

No reduction in LDL-c occurred compared to those who consumed biscuits with plant stanols alone. A stanol-fortified biscuit, as used in the present study, is a new food product intended to be consumed between meals. This new food product form differs from other available commercial products, which are usually in the form of spreads, salad dressings, shortenings for the high fat products, and as yogurt- or soy-based drinks. The test biscuit was formulated to have approximately 5.0 grams of fat with some protein and carbohydrate providing 0.50 MJ (120 kcal) per a biscuit. In the present study, plant stanol was in the ester form, which would improve the solubility of plant stanols into mixed bile salt micelles within the small intestine. These have a moderate fat content that may not trigger gallbladder contraction. Lack of bile acids could interfere with the proper formation of mixed micelles, and thus affect the replacement of cholesterol by plant stanols in the mixed micelles, and in consequence would not inhibit cholesterol absorption [42]. Gall bladder emptying could be induced by 7 grams of fat perfusion in the jejunum [43]. More recently, only 2 grams of fat in emulsion could achieve a marked gall bladder volume change of 27%. However, the fasted gall bladder volume varied remarkably between individuals [44].

In the present study, consuming plant stanol with coffee led to a modest decrease in LDL-c. The coffee consumed by the subjects was mainly iced-coffee, which had considerable energy (1000–1700 kJ/cup upon formula) and can count as a meal. If the energy intake at the time of taking a biscuit was found to be over 920 kJ, it was considered taking a biscuit with a meal. Coffee stimulates cholecystokinin release thus leading to gall bladder contraction [45]. Consequently, the cholesterol-lowering efficacy of plant stanol products may be improved by using food ingredients that stimulate bile release. Daily replacement with plant stanol esters in an ordinary cup of coffee could reduce LDL-c by 12.7% [46]. However, a well-designed future study related to the efficacy of taking coffee with the enriched plant stanol products on lowering cholesterol is needed.

Liver enzymes, serum ALT and GGT, were measured at the beginning and at the end of the intervention period to ensure normal health status. Unexpectedly, 9 (15.0%) and 14 (23.7%) subjects in the control and plant stanol groups, respectively, had elevated baseline liver enzymes. In all subjects, no past history of this health problem was present, based on the screening interview. At the beginning of the intervention, researchers recognized this result when all subjects had already consumed the biscuits for two weeks. Consequently, all subjects continued to consume their biscuits until completing the four-week study period. No signs or symptoms related to liver problems were reported. Serum ALT and GGT concentrations increased significantly from baseline in both plant stanol and control groups both in subjects having normal and in subjects having elevated baseline values. These results indicated that increased liver enzymes did not relate to the addition of plant stanol in the biscuit and had no clinical relevance. Serum ALT levels increased slightly but significantly from baseline in both plant stanol and control groups and the changes did not differ between the groups [47]. The reasons for this increase remain unknown but it is of note that the activity of ALT, GGT, and alkaline phosphatase is unaffected by plant sterol/stanol consumption and remain within the reference range [21].

## 5. Conclusions

This placebo-controlled parallel study demonstrated that the once-a-day intake of 2.0 grams per day of plant stanols as an ester, in a biscuit, significantly reduced serum TC, LDL-c, and LDL/HDL ratios by 4.9%, 6.1%, and 4.3%, respectively, compared to a control group. However, when the biscuit with plant stanols was consumed as part of a meal, a significantly higher reduction in total and LDL cholesterol was achieved compared to biscuit consumption with plant stanols without a meal. Incorporating plant stanols into a biscuit is another possible, convenient, and acceptable way to lower modestly elevated cholesterol concentrations. For optimal efficacy, biscuits should be consumed with a meal as part of a healthy diet.

## Figures and Tables

**Table 1:** Nutritive values of the placebo and plant stanol fortified biscuits.

Nutritional values/biscuit (28 g)	Intervention biscuits		Control biscuits	
	Chocolate	Fruity	Chocolate	Fruity
Energy (kJ)	504	498	519	518
Protein (g)	2.2	2.4	2.3	2.5
Carbohydrates (g)	16.0	15.7	16.4	16.4
Of which sugars	5.7	5.9	5.5	5.5
Fat (g)	4.9	4.8	4.9	4.8
Of which saturated fatty acids	0.8	0.7	0.9	0.8
Of which monounsaturated fatty acids	2.6	2.6	2.5	2.5
Of which polyunsaturated fatty acids	1.3	1.2	1.2	1.3
Of which trans fatty acids	0.1	0.1	0.0	0.0
Plant stanols (g)^*^	1.92	1.92	0.0	0.0
Fiber (g)	1.5	1.8	2.4	2.4

^*^Composition: 83% sitostanol and 17% campestanol.

**Table 2:** Baseline demographic of the mild hypercholesterolemic subjects in the control and plant stanol groups^1^.

	Control (*N* = 60)	Plant stanol		
		Total	Biscuit with meal	Biscuit without meal
		(*N* = 59)	(*N* = 33)	(*N* = 26)
Gender (male/female)	14/46	15/44	12/21	3/23
Age (years)	42.2 ± 9.5	42.9 ± 9.3	41.5 ± 9.0	43.3 ± 9.6
Weight (kg)	61.8 ± 11.8	62.3 ± 14.3	63.6 ± 15.0	60.6 ± 13.3
Height (cm)	158.8 ± 8.1	158.8 ± 8.0	160.2 ± 7.9	157.1 ± 7.8
BMI (kg/m^2^)	24.7 ± 8.5	25.4 ± 11.3	24.7 ± 4.9	24.5 ± 4.6
Cholesterol (mmol/L)				
Total cholesterol	6.01 ± 0.62	6.02 ± 0.78	6.26 ± 0.74	5.72 ± 0.73
LDL-cholesterol	4.13 ± 0.60	4.12 ± 0.75	4.37 ± 0.74	3.81 ± 0.65
HDL-cholesterol	1.59 ± 0.46	1.58 ± 0.34	1.56 ± 0.32	1.62 ± 0.37
Total cholesterol : HDL ratio	4.08 ± 1.20	3.96 ± 0.92	4.17 ± 0.93	3.69 ± 0.83
LDL : HDL ratio	2.84 ± 0.97	2.74 ± 0.80	2.93 ± 0.81	2.49 ± 0.74
Triglyceride	1.40 ± 0.69	1.37 ± 0.56	1.33 ± 0.59	1.42 ± 0.52

^1^Values are means ± SD. To convert mmol/L of cholesterol and triglyceride to mg/dL, multiply by 39 and 89, respectively.

**Table 3:** Energy and nutrient intakes of the mild hypercholesterolemic subjects during the study between control and plant stanol groups^1^.

Nutrients	Control group			Plant stanol group		
	Week 0	Week 2	Week 6	Week 0	Week 2	Week 6
Energy (MJ)	5.6 ± 2.0	5.6 ± 2.0	5.6 ± 2.0	5.7 ± 2.0	5.7 ± 2.0	5.7 ± 2.1
Carbohydrate (g/d)	172.8 ± 60.0	173.0 ± 60.0	174.8 ± 60.5	184.1 ± 76.7	183.2 ± 75.4	180.7 ± 76.2
Protein (g/d)	54.3 ± 24.9	53.3 ± 25.2	54.2 ± 26.3	53.9 ± 21.4	52.4 ± 22.0	52.8 ± 22.6
Fat (g/d)	50.4 ± 25.2	50.2 ± 25.7	50.0 ± 25.9	46.9 ± 21.9	47.4 ± 22.0	47.2 ± 22.0
Dietary fiber (g/d)	9.8 ± 4.6	9.8 ± 4.5	9.9 ± 4.7	9.8 ± 5.2	9.5 ± 5.2	9.6 ± 5.1
Cholesterol (mg/d)	237.5 ± 57.6	235.7 ± 59.4	234.9 ± 60.4	237.9 ± 57.8	238.8 ± 58.5	239.0 ± 59.0
Energy distribution						
Carbohydrate (%)	51.8 ± 9.6	52.2 ± 9.9	52.3 ± 10.5	53.5 ± 10.7	53.5 ± 10.5	53.4 ± 10.2
Fat (%)	32.3 ± 7.8	32.2 ± 8.1	32.0 ± 8.5	30.7 ± 8.3	31.0 ± 8.2	30.9 ± 7.8
Protein (%)	15.9 ± 4.6	15.6 ± 4.5	15.8 ± 4.8	15.8 ± 4.7	15.6 ± 4.5	15.7 ± 4.4

^1^Values are means ± SD. Week 0 (run in); week 2 (baseline); week 6 (end of intervention); no significant difference in all nutrient intakes between two groups at baseline, week 2 and  week 6, by repeated measure analysis of variance.

**Table 4:** Changes in lipid profiles between control and stanol groups (for all subjects and subgroups of subjects who consume a biscuit with and without meal)^1^.

	Control (*N* = 60)	Stanol					
		Total		Biscuit with meal		Biscuit without meal	
		(*N* = 59)		(*N* = 33)		(*N* = 26)	
		Value	Mean difference from control^3^	Value	Mean difference from control^3^	Value	Mean difference from control^3^
Total Cholesterol, mmol/L							
Baseline	6.01 ± 0.62	6.02 ± 0.78	0.02 ± 0.12	6.26 ± 0.74	0.25 ± 0.14	5.72 ± 0.73	−0.29 ± 0.15
4 weeks	6.11 ± 0.69	5.81 ± 0.72^a,∗∗^	−0.30 ± 0.13	5.93 ± 0.70^a^	−0.18 ± 0.15	5.66 ± 0.73^**^	−0.45 ± 0.16
Difference from baseline^2^	0.11 ± 0.47	−0.21 ± 0.63^**^	−0.31 ± 0.10	−0.33 ± 0.55^**^	−0.43 ± 0.11	−0.06 ± 0.71	−0.17 ± 0.15
% change from baseline^2^	2.0 ± 7.7	−2.9 ± 10.6^**^	−4.81 ± 1.70	−4.9 ± 8.4^**^	−6.80 ± 1.72	−0.4 ± 12.8	−2.30 ± 2.68

LDL Cholesterol, mmol/L							
Baseline	4.13 ± 0.60	4.12 ± 0.75	−0.00 ± 0.12	4.37 ± 0.74	0.24 ± 0.14	3.81 ± 0.65	−0.31 ± 0.14
4 weeks	4.14 ± 0.68	3.87 ± 0.64^a,∗∗^	−0.27 ± 0.12	3.97 ± 0.68^a^	−0.17 ± 0.15	3.73 ± 0.57^**^	−0.41 ± 0.15
Difference from baseline^2^	0.01 ± 0.48	−0.26 ± 0.50^**^	−0.27 ± 0.09	−0.40 ± 0.37^**^	−0.41 ± 0.10	−0.08 ± 0.58	−0.09 ± 0.12
% change from baseline^2^	0.7 ± 11.5	−5.4 ± 12.1^**^	−6.06 ± 2.16	−8.9 ± 8.3^**^	−9.61 ± 2.07	−0.9 ± 14.6	−1.56 ± 2.94

HDL Cholesterol, mmol/L							
Baseline	1.59 ± 0.46	1.58 ± 0.34	−0.01 ± 0.07	1.56 ± 0.32	−0.03 ± 0.08	1.62 ± 0.37	0.03 ± 0.10
4 weeks	1.64 ± 0.45^a^	1.63 ± 0.41	−0.01 ± 0.08	1.62 ± 0.39	−0.03 ± 0.09	1.64 ± 0.44	0.00 ± 0.10
Difference from baseline^2^	0.05 ± 0.17	0.04 ± 0.17	−0.01 ± 0.03	0.06 ± 0.13	0.00 ± 0.03	0.03 ± 0.22	−0.03 ± 0.04
% change from baseline^2^	4.1 ± 9.94	2.5 ± 10.8	−1.54 ± 1.90	3.2 ± 8.7	−0.90 ± 2.07	1.8 ± 13.0	−2.35 ± 2.87

Ratio LDL/HDL cholesterol							
Baseline	2.84 ± 0.97	2.74 ± 0.80	−0.10 ± 0.16	2.93 ± 0.81	0.09 ± 0.20	2.49 ± 0.74	−0.35 ± 0.19
4 weeks	2.75 ± 1.01	2.52 ± 0.73^a^	−0.23 ± 0.16	2.60 ± 0.77^a^	−0.15 ± 0.20	2.42 ± 0.67	−0.34 ± 0.19
Difference from baseline^2^	−0.09 ± 0.33	−0.22 ± 0.33^*^	−0.13 ± 0.06	−0.33 ± 0.26^**^	−0.24 ± 0.07	−0.07 ± 0.37	0.01 ± 0.08
% change from baseline^2^	−2.9 ± 11.1	−7.1 ± 12.4^*^	−4.25 ± 2.16	−11.4 ± 8.4^**^	−8.48 ± 2.23	−1.8 ± 14.6	1.12 ± 3.20

Triglyceride, mmol/L							
Baseline	1.40 ± 0.69	1.37 ± 0.56	−0.03 ± 0.12	1.33 ± 0.59	−0.07 ± 0.14	1.42 ± 0.52	0.02 ± 0.15
4 week	1.32 ± 0.60	1.31 ± 0.78	−0.01 ± 0.13	1.33 ± 0.95	0.00 ± 0.16	1.28 ± 0.50	−0.04 ± 0.13
Difference from baseline^2^	−0.08 ± 0.54	−0.06 ± 0.51	−0.01 ± 0.10	−0.01 ± 0.61	0.07 ± 0.12	−0.14 ± 0.32	−0.06 ± 0.11
% change from baseline^2^	3.5 ± 50.9	−3.4 ± 28.4	−6.86 ± 7.57	−0.6 ± 32.2	−4.03 ± 9.79	−7.0 ± 22.7	−10.45 ± 10.42

^1^Values are means ± SD. To convert cholesterol and triglyceride vales to mg/dL, multiply by 38.67 and 88.57, respectively.

^2^Percentage change is based on individual data, (4 weeks-baseline)/baseline × 100.

^3^Mean Difference is the mean difference between intervention and control group, comparison between groups by independent samples *t*-test.

^a^ *P* < 0.05, significant change over time comparing to baseline.

^*^ *P* < 0.01, significantly different from control; ^**^ *P* < 0.05, significantly different from control.

**Table 5:** Change in serum liver function test between control and stanol groups^1^.

	ALT			GGT		
	Baseline	4 weeks	% change^2^	Baseline	4 weeks	% change^2^
Total subjects						
Control (*n* = 60)	19.05 ± 15.93	22.28 ± 13.58^a^	27.9 ± 40.5	30.68 ± 28.07	35.60 ± 36.03^b^	12.1 ± 24.0
Stanol (*n* = 59)	18.47 ± 9.78	22.54 ± 13.62^a^	27.4 ± 57.3	40.32 ± 43.05	47.20 ± 59.23^b^	16.3 ± 48.2
Subjects who have normal baseline liver function test						
Control (n = 51)	15.00 ± 6.73	18.65 ± 9.66^a^	27.4 ± 35.2	21.41 ± 11.10	23.37 ± 12.81^b^	9.3 ± 20.7
Stanol (*n* = 45)	15.86 ± 7.25	20.23 ± 11.58^a^	32.1 ± 63.9	24.23 ± 11.58	29.11 ± 23.51^b^	15.8 ± 44.9

^1^Values are means ± SD. ALT: L-alanne aminotransferase (normal values of ALT: 5–40 U/L); GGT: gamma-glutamyltransferase (normal values of GGT for Male: 10–71 U/L, Female: 6–42 U/L).

^2^Percentage change is based on individual data, (4 weeks-baseline)/baseline × 100.

^a^ *P* < 0.01, significant change over time comparing to baseline of ALT.

^b^ *P* < 0.01, significant change over time comparing to baseline of GGT.
